# Prevalence, awareness, treatment and control of hypertension among adults 50 years and older in Dakar, Senegal

**DOI:** 10.5830/CVJA-2011-039

**Published:** 2012-06

**Authors:** E Macia, P Duboz, L Gueye

**Affiliations:** UMi 3189 Environnement, santé, sociétés, Université Cheikh Anta Diop/CNrs/Université de Bamako/CNrst, Burkina- Faso, Senegal; UMi 3189 Environnement, santé, sociétés, Université Cheikh Anta Diop/CNrs/Université de Bamako/CNrst, Burkina- Faso, Senegal; UMi 3189 Environnement, santé, sociétés, Université Cheikh Anta Diop/CNrs/Université de Bamako/CNrst, Burkina- Faso, Senegal

**Keywords:** hypertension, risk factors, older adults, Senegal

## Abstract

**Background:**

Older adults are disproportionately affected by hypertension, which is an established risk factor for cardiovascular disease. Despite these facts, no study on the prevalence, awareness, treatment and control on arterial hypertension in Senegal has been conducted, specifically among elderly people.

**Methods:**

Five hundred people aged 50 years and older, living in the city of Dakar were interviewed. This sample was constructed using the combined quota method in order to strive for representativeness of the target population.

**Results:**

Prevalence of hypertension was 65.4% in our sample. Half of those suffering from high blood pressure were aware of their problem and among the latter, 70% said they were on treatment. However, of these, only 17% had controlled arterial blood pressure. The only factor associated with awareness, treatment and control of hypertension was the frequency of doctor visits.

**Conclusion:**

Improving follow-up health checks of older adults are necessary to limit the consequences of hypertension in Dakar.

## Abstract

Cardiovascular disease is an emerging problem in sub-Saharan Africa.^1^ In Senegal, mortality associated with such diseases is already over half that related to non-contagious diseases.^2^ Moreover, hypertension is a prime risk factor for cardiovascular disease due to both its widespread prevalence and low control rate among populations,^3^ making hypertension a major public health problem *per se* in sub-Saharan Africa.

Urbanisation and the adoption of a Western lifestyle contribute greatly to the rising incidence of hypertension in sub-Saharan Africa.^4^ Recent studies conducted in the region have shown that hypertension was already as frequent in these cities as it is in developed countries.^5^-^14^ In Dakar, its prevalence among adults 20 years and older was 27.5% in 2009.^15^ These findings are particularly alarming due to the present low rates of detection, treatment and control of hypertension observed in sub-Saharan Africa.^5^

Whether carried out in Western countries or in sub-Saharan Africa, all studies show that the prevalence of arterial hypertension rises drastically with age and that the elderly are the population segment the most at risk.^7^,^16^ In Dakar, again according to the previously cited study, nearly 70% of adults 50 years and older are believed to suffer from hypertension.^15^ Despite this evidence, and to our knowledge, no study pertaining to the awareness, treatment and control of arterial hypertension in sub-Saharan Africa has been specifically conducted among the elderly. Most research conducted in this geographic area considers older people as a non-specific and homogenous population category (the ‘50 years and older’ for example). Yet, studies carried out in both developed and developing countries demonstrate clear evolution in the prevalence, awareness, treatment and control of hypertension during the aging process.^17^-^19^

The aims of this study were therefore to (1) assess the prevalence, awareness, treatment and control of hypertension in the population aged 50 years and older living in the city of Dakar; (2) identify factors associated with hypertension, and also its awareness, treatment and control.

## Methods

This study was conducted from January to June 2009 on a sample of 500 individuals. The sample was constructed using the quota method (cross-section by age, gender and town of residence) in order to strive for representativeness of the population 50 years and older living in the city of Dakar. Data from the Agence Nationale de la Statistique et de la Démographie dating from the last census (2002) were used to this end. The quota variables used were gender (male/female), age (50–59, 60–69, 70 years and older) and town of residence.

The towns were grouped into the four districts making up the city of Dakar: Plateau-Gorée (five towns), Grand Dakar (six towns), Parcelles Assainies (four towns) and Almadies (four towns). This method requires building up a sample that follows the proportions observed in the general population: for example, according to the last census, men aged 50–59 years living in the town of Medina (district of Plateau-Gorée) represented 2.4% of the population of 50 years and older living in the city of Dakar. The sample was constructed so as to reflect this proportion and included 12 men 50–59 years old living in this town.

For each town, four investigators (PhD students in the departments of Medicine and Pharmacy) started out from different points each day to measure and interview individuals in Wolof or French in every third home. Investigators had a set number of individuals to interview (women and men 50–59 years, 60–69 years, and 70 years and over in each town) to meet the quotas. Only one person was selected as a respondent in each home.

The objective of this bio-anthropological survey was to carry out a holistic study on aging in the city of Dakar. To do so, face-to-face guided interviews based on a questionnaire were used to collect the data required for the study. These interviews were followed by a physical examination that involved taking blood pressure and anthropometric measurements.

## Study definitions and measurements

Blood pressure was measured twice for each participant in the course of a single visit. The first measurement was taken mid-way through the interview, just after the questions related to individual health. The second measurement was taken at the end of the questionnaire, after about 15–20 minutes’ rest. These measurements were taken by medical and pharmacy students in Dakar, using an Omron® M3 Intellisense device validated by the International Protocol.^20^ The mean of the two measurements was used for the analyses.

In accordance with the Seventh Report of the Joint National Committee of Prevention, Detection, Evaluation, and Treatment of High Blood Pressure, individuals with systolic blood pressure ≥ 140 mmHg and/or diastolic blood pressure ≥ 90 mmHg and/or who reported the current use of antihypertensive medication were considered to be suffering from high blood pressure.^21^

Weight was measured using a digital scale (accuracy of 100 g) with subjects dressed in minimum clothing and barefoot. To measure height, the subject was asked to stand ‘at attention’, arms at the sides, heels together and without shoes. Following World Health Organisation recommendations, body mass index (BMI) was calculated by dividing weight (kg) by the square of the height (m^2^). Overweight was defined as 25 ≤ BMI < 30 kg/m^2^; obesity corresponded to a BMI of ≥ 30 kg/m^2^.^22^

Given the large proportion of people who had not visited a doctor in the year preceding the interview (48%), the frequency of doctor visits was split into two groups, as in the study conducted by the hypertension study group in India and Bangladesh.^17^ Therefore, people who had not visited a doctor in the year preceding the interview were distinguished from those who had seen a doctor at least once during the year.

Among the socio-demographic data collected during the interviews, four variables were taken into account for this study: age, gender, educational level and marital status. Three age groups were defined: 50–59, 60–69 and 70 years and over. Gender was coded as follows: 1 for women, 0 for men. Three levels of education were defined: none, one to eight years of schooling, more than eight years of schooling. Marital status was coded as follows: married = 0, other = 1.

## Statistical analysis

To answer our research questions, we used Chi-square tests and logistic regressions. The software used for the statistical analysis was PASW Statistics 18.

## Results

The socio-demographic characteristics of our population sample and the descriptive results regarding frequency of doctor visits and BMI are presented in Table 1. Men were better educated and less often overweight or obese than women. On the other hand, more women had visited a doctor in the year preceding the interview.

**Table 1.** Characteristics Of The Sample (*n* = 500)

*Variable*	*Category*	*Total, n (%)*	*Men, n (%)*	*Women, n (%)*	*Analysis*
Age (years)	50–59	268 (53.6)	144 (54.7)	124 (52.3)	Chi^2^ (2 df) = 0.41; NS
	60–69	136 (27.2)	71 (27)	65 (27.4)	
	≥ 70	96 (19.2)	48 (18.3)	48 (20.3)	
Educational level	None	228 (45.6)	97 (36.9)	131 (55.3)	χ^2^ (2 df) = 29.46; *p* < 0.001
	1–8 years	186 (37.2)	100 (38.0)	86 (36.3)	
	≥ 9 years	86 (17.2)	66 (25.1)	20 (8.4)	
Marital status	Married	372 (74.4)	234 (89)	138 (58.2)	χ^2^ (1 df) = 61.87; *p* < 0.001
	Not married	128 (25.6)	29 (11)	99 (41.8)	
Doctor visits in previous year	0	240 (48)	141 (53.6)	99 (41.8)	χ^2^ (1 df) = 7.00; *p* < 0.01
	≥ 1	260 (52)	122 (46.4)	138 (58.2)	
BMI (kg/m^2^)	< 25	231 (46.2)	149 (56.7)	82 (34.6)	χ^2^ (1 df) = 24.40; *p* < 0.001
	≥ 25	269 (53.8)	114 (43.3)	155 (65.4)	

**Fig. 1.**
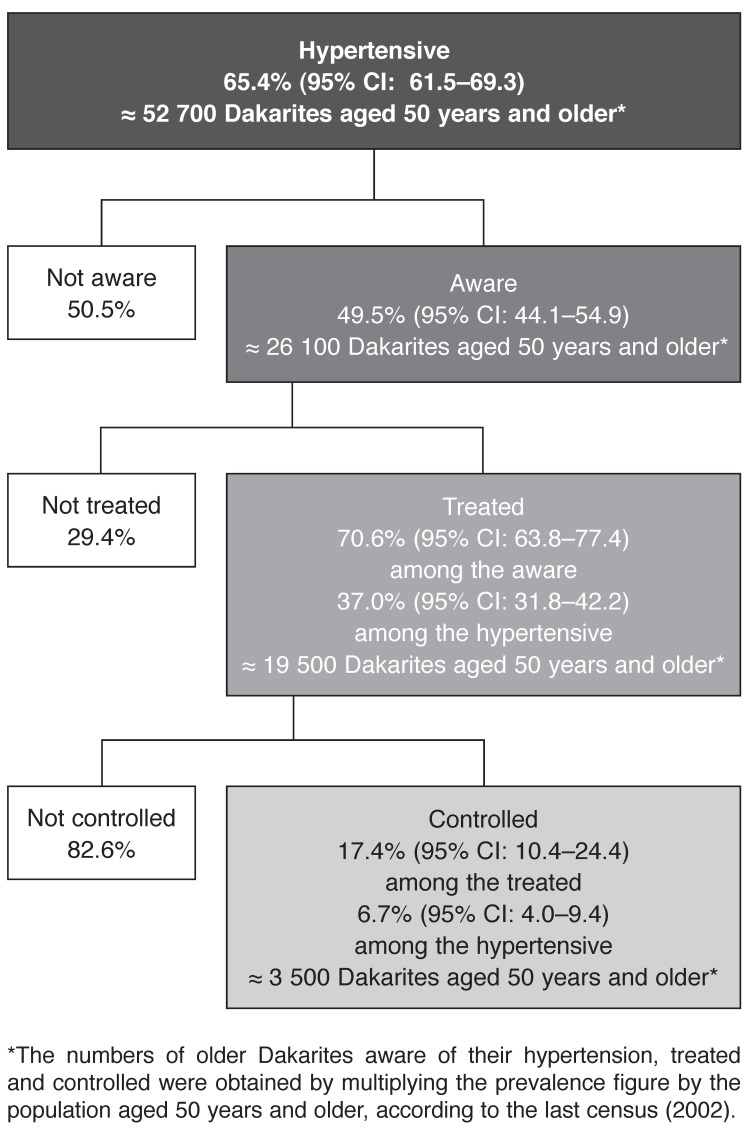
Prevalence, awareness, treatment and control of hypertension in the population of Dakar aged 50 years and older.

In our sample, the prevalence of hypertension was 65.4% [95% confidence interval (CI): 61.5–69.3). Nearly half of the individuals suffering from hypertension were aware of their health problem, and 70% of the informed people reported being treated for hypertension. Therefore, 37% (95% CI: 31.8–42.2) of the people suffering from hypertension were treated. However, among people reporting they were treated for hypertension, only 17.4% had controlled hypertension; i.e. 6.7% (95% CI: 4.0–9.4) of the hypertensives (Fig. 1).

Bivariate analyses showed that hypertension increased steadily with age in our sample, from 58% among those 50–59 years old to 76% among those 70 years and older. These analyses also showed that overweight or obese individuals were more often affected by hypertension than others (70.6 vs 59.3%, respectively). On the other hand, gender and marital status were not significantly associated with hypertension (Table 2). The bivariate results were confirmed using logistic regression analysis (Table 3).

**Table 2.** Factors Associated With Hypertension, Awareness, Treatment And Control

		*Prevalence (n = 500)*		*Awareness (n = 327)*		*Treatment among hypertensives (n = 327)*		*Treatment among aware (n = 171)*		*Control among hypertensives (n = 327)*		*Control among treated (n =121)*	
*Variable*	*Category*	*%*	*Analysis*	*%*	*Analysis*	*%*	*Analysis*	*%*	*Analysis*	*%*	*Analysis*	*%*	*Analysis*
Total		65.4		49.5		37		70.6		6.7		17.4	
Gender	Men	63.9	χ^2^ (1 df) = 0.57; NS	36.3	χ^2^ = 24.20; *p* < 0.001	27.4	χ^2^ = 13.72; *p* < 0.001	71.4	χ^2^ = 0.34; NS	3.6	χ^2^ = 5.49; *p* < 0.05	13	χ^2^ = 0.96; NS
	Women	67.1		63.5		47.2		70.1		10.1		20	
Age (years)	50–59	58.2	χ^2^ (2 df) = 13.60; *p* < 0.001	42.3	χ^2^ (2 df) = 8.65; *p* < 0.05	30.8	χ^2^ = 6.42; *p* < 0.05	66.7	χ^2^ = 0.94; NS	6.4	χ^2^ = 0.05; NS	18.8	χ^2^ = 0.33; NS
	60–69	72.1		51		38.8		74		7.1		18.4	
	≥ 70	76		63		47.9		72.9		7.8		14.3	
Educational level	None	68.9	χ^2^ (2 df) = 3.91; NS	52.2	χ^2^ = 6.59; *p* < 0.05	34.4	χ^2^ = 2.23; NS	62.8	χ^2^ = 6.27; *p* < 0.05	8.3	χ^2^ = 1.32; NS	24.1	χ^2^ = 3.07; NS
	1–8 years	65.1		52.9		42.1		75.8		5.8		11.8	
	≥ 9 years	57		32.7		32.7		88.9		4.1		12.5	
Marital status	Married	66.1	χ^2^ (1 df) = 0.34; NS	43.9	χ^2^ = 12.63; *p* < 0.001	32.9	χ^2^ = 7.08; *p* < 0.01	71.4	χ^2^ = 0.11; NS	6.1	χ^2^ = 0.63; NS	18.5	χ^2^ = 0.23; NS
	Not married	63.3		66.7		49.3		69		8.6		15	
Doctor visits in previous year	0	62.9	χ^2^ (1 df) = 1.26; NS	35.1	χ^2^ = 23.41; *p* < 0.001	19.2	χ^2^ = 38.12; *p* < 0.001	53.7	χ^2^ = 10.87; *p* < 0.001	2	χ^2^ = 10.05; *p* < 0.01	19.6	χ^2^ = 1.31; NS
	≥ 1	67.7		61.9		52.3		78.4		10.8		10.3	
BMI (kg/m^2^)	< 25	59.3	χ^2^ (1 df) = 7.03; *p* < 0.01	46	χ^2^ = 1.19; NS	34.3	χ^2^ = 0.74; NS	69.7	χ^2^ = 0.04; NS	8	χ^2^ = 0.64; NS	23.4	χ^2^ = 1.96; NS
	≥ 25	70.6		52.1		38.9		71.2		5.8		13.5	

**Table 3.** Adjusted Odds Ratios For Hypertension, Awareness, Treatment And Control

		*Hypertension (n = 500)*		*Awareness (n = 327)*		*Treatment among hypertensives (n = 327)*		*Treatment among aware (n = 171)*		*Control among hypertensives (n = 327)*		*Control among treated (n = 121)*	
*Variables*	*Categories*	*OR*	*IC (95%)*	*OR*	*IC (95%)*	*OR*	*IC (95%)*	*OR*	*IC (95%)*	*OR*	*IC (95%)*	*OR*	*IC (95%)*
Gender (Men)	Women	1.01	0.66–1.56	2.4**	1.41–4.07	2.3**	1.31–4.06	1.45	0.59–3.56	2.79	0.92–8.47	1.56	0.41–5.9
Age (50–59 years)	60–69	1.94**	1.22–3.07	1.44	0.82–2.54	1.62	0.89–2.94	2.16	0.89–5.26	1.03	0.35–3.05	0.73	0.21–2.6
	≥ 70	2.54**	1.45–4.44	2.15*	1.11–4.17	2.24*	1.14–4.4	2.23	0.85–5.86	0.96	0.27–3.42	0.66	0.16–2.71
Educational level (≥ 9 years)	None	1.28	0.73–2.23	1.72	0.8–3.69	0.73	0.33–1.61	0.2	0.04–1.07	1.66	0.32–8.56	2.14	0.34–13.54
	1–8 years	1.23	0.71–2.14	2.15*	1.01–4.6	1.36	0.63–2.94	0.49	0.09–2.56	1.31	0.24–7.06	0.98	0.15–6.29
Marital status (Married)	Not married	0.81	0.51–1.28	1.48	0.8–2.75	1.13	0.61–2.1	0.54	0.22–1.32	0.94	0.32–2.77	0.9	0.25–3.27
BMI (< 25 kg/m^2^)	≥ 25 kg/m^2^	1.86**	1.24–2.79	1.23	0.73–2.06	1.13	0.66–1.91	0.94	0.44–2.03	0.7	0.27–1.81	0.62	0.22–1.8
Doctor visit in previous year (≥ 1)	0			0.37***	0.23–0.6	0.24***	0.14–0.41	0.32**	0.15–0.69	0.17**	0.05–0.6	0.32	0.07–1.43

*p < 0.05; **p < 0.01; ***p < 0.001.

Aside from BMI, using bivariate analyses, all factors studied were associated with awareness of hypertension. Women, the older and unmarried individuals were more often informed of this problem than men, younger people and married individuals. Likewise, many more individuals who had seen a doctor at least once in the year preceding the interview were aware of their hypertensive condition than those who had not seen a doctor during this period. Lastly, and more surprisingly, people who had had at least nine years of schooling were less often aware of their hypertensive status than the less educated (Table 2). Most of these results were controlled using logistic regression analysis and only marital status was not significantly associated with awareness of hypertension (Table 3).

Multivariate analysis showed that among hypertensives, women, older adults, and those who had seen a doctor during the preceding year more often reported taking treatment than men, younger people, and those who had not seen a doctor during the previous year, respectively (Table 3).

The results for the sub-sample of individuals who were aware of their hypertension problem were quite different. In this logistic regression analysis, only the frequency of doctor visits was significantly associated with treatment of hypertension (Table 3).

Among the hypertensives, on multivariate analysis, only the frequency of doctor visits was associated with control of hypertension (Table 3). Therefore, people having seen a doctor during the preceding year more often had controlled hypertension than those who had not seen a doctor the previous year. However, among treated individuals, no variable was associated with control of hypertension (Table 3).

## Discussion

The prevalence of hypertension in our population sample corresponded with that observed among older people in other sub-Saharan African cities^5^-^14^ or in other developing countries such as India and Bangladesh.^17^ In Dakar, two out of three people 50 years and older suffered from arterial hypertension, a disease that has now become a major public health concern in the Senegalese capital.

In keeping with what has been observed among other populations, aging and problems of overweight and obesity were associated with hypertension.^23^,^24^ However, this was not the case with educational level. This observation seems to indicate that the Dakar population is currently in an advanced stage of epidemiological transition. This process is characterised by a transfer of risk factors for chronic illnesses from the better-educated individuals in the early stages of the process to the less educated at the end of the transition.^25^

The rate of awareness of hypertension among the hypertensives, approximately 50%, corresponds with that observed among the elderly living in other developing countries.^17^ This rate is, however, much lower than that noted in the West, where over two-thirds of older hypertensives are aware of the problem.^18^,^19^

If the ‘rule of halves’^26^ remains valid here, it nevertheless conceals great disparities, especially between men and women. As with most developing populations, women were more often informed on their problem of hypertension than men.^27^ However, the reasons for this association remain poorly understood.^17^ In fact, it may appear surprising in Senegal, where male domination over women is taken for granted.^28^

The Demographics and Health Survey conducted in 2005 indicated for instance that scarcely 12% of married women made their own decisions about their personal healthcare spending, whereas for 67% of them, only their spouse made such decisions.^29^ However, in Senegal, it is primarily women who take care of the health of members of the household, accompanying their daughters, daughters-in-law and grandchildren to healthcare institutions. This might explain both their more frequent visits to these institutions and their greater monitoring of hypertension.

Unlike the results noted for elderly German and American populations,^18^,^19^ awareness of hypertension rises with age among the elderly in Dakar. Therefore the probability of having been identified as hypertensive rises with age. More surprisingly, we have seen that people with a higher educational level were often less informed on their hypertension than those with an average educational level. This result runs contrary to all research conducted on the subject, which generally demonstrates the opposite.^30^ More research is required to understand this specificity, but it could be that education does not have the same implications for health management in Dakar as in developed countries. Nevertheless, it is not surprising to note that the factor most strongly associated with awareness of hypertension was the frequency of doctor visits.

More than 70% of individuals aware of their hypertension reported taking treatment, which seems well above the rule of halves. This theoretically encouraging statistic should, however, be discussed in light of the results associated with control of hypertension. Fewer than 17% of the people who reported being treated actually had controlled hypertension, i.e. 6.7% of hypertensives.

A study conducted in Ghana could help explain why the hypertension control rate was so low among the elderly in Dakar. According to this study, 93% of the people treated for hypertension did not comply with their medical prescriptions, usually due to the high cost of medication.^31^ The same observation seems to hold true in Dakar where the price of medication is disproportionate to average expenditure per person per day, i.e. 1 224 FCFA (≈ 2.7 dollars).^32^

However, another explanation could be advanced. According to Salem, treatment of chronic disease is generally misunderstood. In Dakar, when a disease is identified, it is believed it should be ejected as a foreign body.^33^ The notion of chronic illness goes against this conception, which could explain the low level of compliance with treatment.

Since pharmacological treatment of hypertension is the consequence of its detection by healthcare personnel, factors associated with treatment among hypertensives were the same as those associated with awareness of this health problem, i.e. frequency of doctor visits, gender and age. Among these factors, only the frequency of doctor visits was significantly associated with the control of hypertension. Therefore it was the only factor investigated that was associated with awareness, treatment and control of hypertension in this study. This result highlights the absolute necessity of improving the follow-up health checks of older adults to minimise the consequences of hypertension in Dakar.

## Strengths and limitations of the study

This research was, to our knowledge, the first study conducted specifically on hypertension among the elderly in sub-Saharan Africa. In years to come, the elderly in developing countries will represent the majority of older people on the planet.^34^ Therefore it is necessary to understand the prevalence of hypertension among these populations, as well as the rates of awareness, treatment and control of the disease, in order to combat this burden more effectively and in a more appropriate manner.

This study has several limitations. As in many studies, arterial blood pressure was measured twice during a single visit, which may have led to overestimation of the prevalence of hypertension. Furthermore, the treatment rate of hypertension was assessed solely by individual self-reporting. Verification of the actual presence of medication in the home might have limited the bias associated with these declarations.

## Conclusion

The results of this study have several public health implications. Firstly, two-thirds of the Dakar elderly suffer from hypertension, and this disease therefore constitutes a major public health concern in the Senegalese capital. Detection could be considerably improved given that only 50% of those suffering from high blood pressure were aware of this problem. Nearly three-quarters of the people informed on their condition reported being treated, which is an encouraging statistic in a developing country. However, compliance with these treatments appears particularly problematic, given that fewer than 20% of individuals treated had controlled hypertension. It is likely that the high cost of pharmacological treatment when compared to income was responsible for the low rate of compliance with these treatments.

One of the factors studied was associated with awareness, treatment and control of hypertension: the frequency of doctor visits. This result highlights the absolute necessity to improve follow-up health checks of older adults to minimise the consequences of hypertension in Dakar.
